# Stearoyl-CoA desaturase 1 expression is downregulated in liver and udder during *E. coli* mastitis through enhanced expression of repressive C/EBP factors and reduced expression of the inducer SREBP1A

**DOI:** 10.1186/s12867-016-0069-5

**Published:** 2016-07-20

**Authors:** Tianle Xu, Xiangzhen Shen, Hans-Martin Seyfert

**Affiliations:** Leibniz Institute for Farm Animal Biology, Institute for Genome Biology, Wilhelm-Stahl-Allee 2, 18196 Dummerstorf, Germany; College of Veterinary Medicine, Nanjing Agricultural University, Weigang 1, Nanjing, 210095 People’s Republic of China

**Keywords:** Cattle, C/EBP, Fat metabolism, Liver, Mastitis, SCD1, Systemic reaction, Udder

## Abstract

**Background:**

Stearoyl-CoA desaturase 1 (SCD1) desaturates long chain fatty acids and is therefore a key enzyme in fat catabolism. Its synthesis is downregulated in liver during illnesses caused by high levels of circulating lipopolysaccharide (LPS). SCD1 expression is known to be stimulated under adipogenic conditions through a variety of transcription factors, notably SREBP1 and C/EBPα and −β. However, mechanisms downregulating SCD1 expression during illness related reprograming of the metabolism were unknown. *Escherichia coli* elicited mastitis is an example of such a condition and was found to downregulates milk and milk fat synthesis. This is in part mediated through epigenetic mechanisms. We analyzed here mechanism controlling SCD1 expression in livers and udders from cows suffering from experimentally induced *E. coli* mastitis.

**Results:**

We validated with RT-qPCR that SCD1 expression was reduced in these organs of the experimental cows. They also featured decreased levels of mRNAs encoding SREBP1a but increased levels for C/EBP α and −β. Chromatin accessibility PCR (CHART) revealed that downregulation of SCD1 expression in liver was not caused by tighter chromatin compaction of the SCD1 promoter. Reporter gene analyses showed in liver (HepG2) and mammary epithelial (MAC-T) model cells that overexpression of SREBP1a expectedly activated the promoter, while unexpectedly C/EBPα and −β strongly quenched the promoter activity. Abrogation of two from among of the three C/EBP DNA-binding motifs of the promoter revealed that C/EBPα acts in *cis* but C/EBPβ in *trans*. Overexpressing truncated C/EBPα or −β factors lacking their repressive domains confirmed in both model cells the direct action of C/EBPα, but not of C/EBPβ on the promoter.

**Conclusions:**

We found no evidence that epigenetic mechanism remodeling the chromatin compaction of the SCD1 promoter would contribute to downregulate SCD1 expression during infection. Instead, our data show for the first time that C/EBP factors may repress SCD1 expression in liver and udder rather than stimulating as it was previously shown in adipocytes. This cell type specific dual and opposite function of C/EBP factors for regulating SCD1 expression was previously unknown. Infection related activation of their expression combined with downregulated expression of SREBP1a explains reduced SCD1 expression in liver and udder during acute mastitis.

**Electronic supplementary material:**

The online version of this article (doi:10.1186/s12867-016-0069-5) contains supplementary material, which is available to authorized users.

## Background

Infection of the udder causing mastitis is a frequent disease of lactating dairy cows [1]. Gram-negative pathogens, such as *Escherichia coli* (*E. coli*) frequently elicit clinical symptoms [2, 3]. These include reduced milk synthesis and a complete shutdown of casein synthesis, mediated in part through chromatin remodeling at a doublet STAT5-transcription factor binding site of the casein promoters [4]. The often generalized inflammatory response during *E. coli* mastitis [5, 6] is conceivably caused through liberation and systemic circulation of lipopolysaccharide (LPS), that major cell envelop component of Gram-negative bacteria. LPS binds to the Toll-like-receptor 4 (TLR4) [7, 8]. Ligand binding triggers its signaling to ultimately activate the NF-κB complex of transcription factors. These in turn will act as a master switch to regulate a wealth of immune genes [9], including pro-inflammatory cytokines such as tumor necrosis factor alpha (TNF-α), interleukin-1 (IL-1) and -6 (IL-6). All these factors are rapidly produced during udder infection—or even during sterile mastitis having experimentally elicited through LPS infusion into the udder—and eventually initiate in the liver the expression of genes contributing to the acute phase response (APR) [10–13]. Hence, systemically circulating LPS derived from mastitis or other diseases such as the subacute acidosis in ruminants (SARA) [14] may eventually reprogram liver metabolism [15].

It was recently observed that the expression of the stearoyl-CoA desaturase 1 (SCD1) is down regulated in the liver of dairy cows and mammary gland of dairy goats during SARA, conceivably triggered through systemically circulating high levels of endogenous LPS derived from rumen [16–19]. The SCD1 enzyme is rate limiting for the formation of monounsaturated fatty acids by introducing a double bond on the position of Δ9. This reaction determines the formation of triglyceride and cholesterol esters in liver and mammary gland [20], hence a key processes in fat synthesis influencing also the overall milk yield [21]. SCD1 plays also a role in modulating inflammation and stress [22]. This may in part be conveyed through epigenetic mechanisms, since the SCD1 activity was found to correlate with the degree of CpG island DNA-methylation at the promoters of relevant immune genes [23]. Hormonal and nutrient factors are known to regulate SCD1 expression in several species including man and mouse [24, 25]. These multifactorial controls are mediated through a variety of different transcription factors [25]. Fine mapping and functional analyses of the SCD1 promoter revealed in mouse, human and cattle, that the sterol regulatory element-binding transcription factors 1 (SREBP1; see [26] for a review) and the CCAAT/enhancer-binding protein alpha (C/EBPα) were found to be pivotal to cooperatively activate the expression of this gene in adipocytes and other cells, in particular during adipogenic differentiation and stimulation [27–32].

The C/EBP family of transcription factors comprises six members (C/EBPα, -β, -γ, -δ, -ε, -ζ). They all feature several domains for factor interaction and activation or repression of transcription [33, 34]. C/EBPα and −β both have a DNA binding region and a leucine zipper for factor dimerization. The −α factor features in addition two activation domains (AD) and one attenuator domain. Three activation domains and two repressive domains (RD) are known to mediate the function of C/EBPβ [35, 36]. C/EBPα and −β are indispensable for adipocyte differentiation and fatty acid synthesis (see [37] for a review). Their expression is quantitatively differentiated in a tissue specific fashion and differentially regulated during the acute phase response [38]. C/EBPα is most prominently expressed in liver and contributes to maintaining lipid homeostasis [34]. C/EBPβ (also known as NF-IL6) is the most abundant family member in the mammary gland. It is a key factor for regulating immune functions in the udder [39] but also involved in controlling fatty acid synthesis in the mammary epithelial cells (MEC) not least through regulating there the expression of the acetyl-CoA carboxylase-alpha (ACACA), the rate limiting enzyme for de novo synthesis of fatty acids ([40] and references therein). C/EBPα and −β are both known to physically bind to—and cooperatively function with—the NF-κB p50 factor [41–44].

No information is available about mechanisms down regulating SCD1 expression in infected organs and tissues. We wondered if epigenetic mechanisms operating during acute *E. coli* mastitis might be involved. We exploited tissues having previously been collected during a trial of experimentally induced clinical *E. coli* mastitis in cows [6]. It was shown in such infection experiments, that epigenetic mechanisms are contributing to the *E. coli* mastitis related acute shut down of casein synthesis [4] but also to the upregulation of infection related genes in the livers of the same cows during the systemic reaction accompanying acute mastitis [45]. Chromatin remodeling may modulate recruitment of transcription factors to their target promoters [46] and is therefore a crucial and diagnostic marker for the operation of such regulatory mechanisms. However, our analyses in the current study revealed no evidence for mastitis related chromatin remodeling of the SCD1 promoter. Rather, we found that downregulated SREBP1 and—surprisingly—upregulated C/EBPα and −β expression explains the infection related shut down of SCD1 expression in the liver during *E. coli* mastitis. We unambiguously prove in models for liver and mammary epithelial cells that here the C/EBP factors −α and −β are repressors rather than enhancers of SCD1 expression as was commonly assumed based on data derived from adipocytes.

## Methods

### Tissues, RNA extraction and chromatin preparation

Tissue samples had been collected during a trial of experimental *E. coli* mastitis. The infection trial (including its ethical approval) and the analysis based on global transcriptome profiling have previously been reported [6]. Briefly, the experimental setting involved infecting one udder quarter of healthy mid lactating Holstein–Friesian heifers with 500 CFU of *E. coli* strain 1303, leaving 3 quarters as controls. The cows were culled 24 h after infection. All infected quarters had developed mastitis with clinical signs. The trial included to sample also control tissues from age- and lactation stage-matched entirely healthy “gold standard” cows. Livers and udders were excised immediately after culling and within <5 min small cubes (0.5 cm diameter) were immediately snap frozen in liquid nitrogen and stored herein. Fifty mg of the tissue were powdered in a mortar under liquid nitrogen and RNA was extracted with TRIZOL (invitrogen) according to the manufacturer’s instructions. Chromatin was prepared from 100 mg of powdered liver samples, essentially as described [45].

### Chromatin compaction assay

**Fig. 1:**
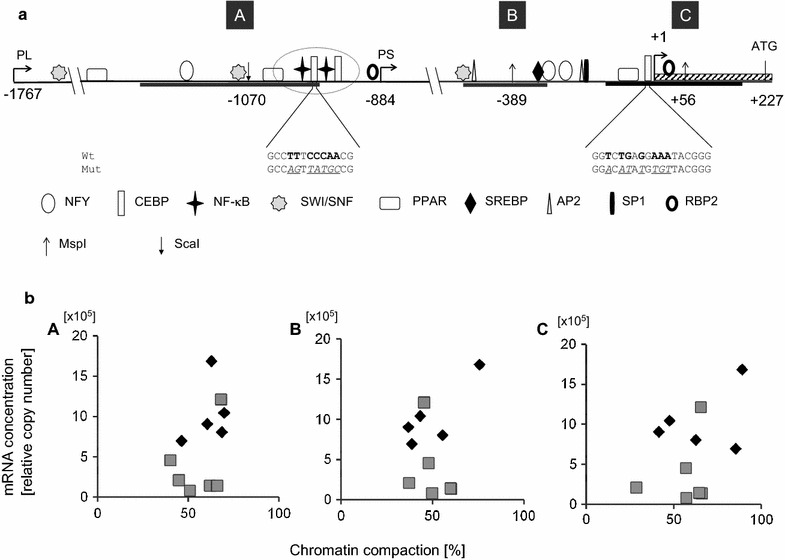
Map of SCD1 promoter in cattle and determination of SCD1 expression and chromatin compaction. **a** The tsp as identified in 5′-RACE experiments is indicated (+1; *hatched box*, exon 1) and positions of selected putative transcription factor bindings sites (in silico analysis; symbols explained in legend below) are mapped relative to the tss. Encircled is a doubled site of overlapping C/EBP and NF-κB binding sites, at around position −1020. *Short kinked arrows* indicate the 5′-ends of the long and short promoter segments (PL, PS, respectively) used in reporter gene assays. Nucleotide sequences of two C/EBP binding sites are given below the map (core nucleotides in *bold face letters*). The mutated variants hereof are shown below (sequence alterations *underlined*). *Gray bars label* the three areas (A, B, C) having been analyzed in CHART-PCR exploiting the restriction enzyme cutting sites as indicated by the *vertical arrows*. **b** The degree of chromatin compaction in three different promoter areas (areas A, B, C), as determined in CHART-PCR (abscissa, expressed as percent of undigested control) is plotted against the levels of SCD1 mRNA concentration (ordinate) from healthy control cows (*grey squares*) or cows having experimentally been infected for 24 h with *E. coli* (*diamonds*). A Distal area A, analyzed with primers pfA/prA and S*ca*I digestion; B Area around the SREBP1 binding site, analyzed with primers pfB/prB and M*sp*I digestion. C Proximal area C, analyzed with primers pfC/prC and M*sp*I digestion. The degree of chromatin compaction did not significantly differ between livers from both groups of cows and there was no correlation between chromatin compaction and the level of gene expression

Chromatin compaction analysis was performed by chromatin accessibility by real time PCR (CHART-PCR). The technique involves preparation of chromatin from nuclei having freshly been isolated from the stored tissue and conducting a limited restriction digestion of the chromatin. Subsequently, the DNA is purified and the relative amount of undigested DNA from the target area is determined in quantitative PCR assays. This fraction represents the protected chromatin. We conducted the assays according to our previous publication [45] with some modifications. Briefly, 4 μl of the nuclear suspension were added into digestion buffer containing 40 U of the restriction endonuclease S*ca*I (for analyzing promoter area A; see map in Fig. 1a) or 20 U of M*sp*I (for analysis of areas B and C), and incubated at 37 °C for 2 h. Subsequently, DNA was purified (High Pure PCR Product Purification Kit; Roche) and quantified with a NanoDrop spectrophotometer. Seventy-five nanograms were used as DNA input for every measuring point of the CHART-PCR and analysed in quantitative real-time PCR with the LightCycler instrument and SYBR Green I kit (both from Roche). Control samples were similarly treated but no restriction enzyme was added. These data represented the input control indicating 100 % protected chromatin. Primers are listed in the Additional file 1: Table S1.

### RT-qPCR

Primers for RT-qPCR are listed in Additional file 1: Table S1A. For cDNA generation, 5 μg of total RNA were transcribed in reverse using SuperScript II (invitrogen) and the general conditions as previously described [47]. The cDNA was purified with the High Pure Purification kit (Roche) and subsequently diluted to a final effluent volume of 100 µl. For each measuring point, aliquots of 5 µl were supplemented with amplification primers (20 pM) and the components of the Sybr Green I kit for PCR reaction. Amplification cycles consisted of an initial denaturation (95 °C, 10 min) followed by 40 cycles of annealing (60 °C, 30 s), elongation (72 °C, 1 min), fluorescence acquisition (83 °C, 5 s) and melting (95 °C, 30 s). Finally, the melting curves of the products were recorded. Quality of all PCR products was also visualized and validated in agarose gel electrophoresis. Relative copy numbers were calculated based on external standard curves obtained from dilution series (10^6^–10 copies) of the cloned amplicons. Their authenticity had been verified by sequencing. Copy numbers were normalized relative to those of the “house keeping gene” SFRS4 (splicing factor, serine/arginine rich 4). Its validity as reference for the normalization of gene expression in liver samples has previously been documented [48] and we reconfirmed it for our samples (Additional file 1: Table S1). Primers are listed in the Additional file 1: Table S2A.

### Reporter gene construction

Primers for cloning SCD1 promoter constructs are listed in Additional file 1: Table S2B. PCR was used to amplify the wild type (wt) promoter of SCD1 using bovine genomic DNA as a template. The forward and reverse primers introduced K*pn*I and H*ind*III restriction sites, respectively. We amplified a long promoter segment (PL; forward primer pf1, binding at –position −1767) and a shorter truncated promoter segment (PS; forward primer pf2, binding at position −884) with the common reverse primer pr2 (binding at position +207). PCR products were first cloned into pGEM-Teasy (Promega), validated by sequencing and the inserts were then excised via the artificially introduced restriction sites. They were subsequently cloned into the promoter-less pGL3-basic firefly luciferase expression vector (Promega). All clones were validated by sequencing.

Mutations of C/EBP binding site on the SCD1 promoter constructs were generated by fusion PCR-mediated mutagenesis [49] using the long reporter gene construct (PL) as a template. Two overlapping fragments were amplified for mutating the distal C/EBP binding site. Generation of the upstream fragment used pf1 as forward primer combined with the reverse primer mpr4 featuring the mutated C/EBP motif. The overlapping downstream fragment used the mutation containing forward primer mpf3 and downstream the reverse primer pr2. Both products were purified and recovered via agarose gel electrophoresis. Annealing of both fragments was achieved by combining both fragments in a single PCR assay (without adding any primers) and cycling the mixture 5 times using the program: Pre-denaturation (94 °C, 5 min), followed by 5 touch-down cycles each lowering the annealing temperature from an initial 65 by 1 °C per cycle, 1 min; 72 °C, 4 min; 95 °C, 1 min. The mutated full length promoter fragment was recovered by next adding primers pf1 and pr2 and cycling another 30 times, using 60 °C as annealing temperature. The final product was purified via gel electrophoresis, cloned into pGEM-Teasy and sequenced. Primers mpf5 and mpr6 were devised for the mutation of proximal C/EBP site and applying the same PCR strategy as described above using the short promoter clones as a template. The PCR products were cloned first into pGEM-T easy (promega) and validated by sequencing. Correct amplicons were cloned into the pGL3-basic vector designating the mutated constructs as PL-MD and PS-MP and named long promoter with distal mutation. For muting both C/EBP sites we used clone PL-MD as a template, mutated the proximal site just as described above and designated the final clone as PL-MB (for both sites mutated).

### Expression constructs for transcription factors

All vectors expressing transcription factors have previously been described including the validation of their expression with western-blots and/or via super-shifting with specific antibodies in EMSA analyses; those of the series of NFY factors from cattle (NFY-A, -B, -C) in [49]; those expressing the various C/EBP factors from cattle and their truncated DN-variants in [40]; those expressing the NF-κB p50 and −p65 factors from cattle in [39]. The vector expressing the human SREBP1a factor was a kind gift of Dr. J. Mao (Baylor College, Houston, TX, U.S.A.).

### Cell culture, transfection and determination of the reporter-gene activity

Reporter gene constructs for evaluating promoter activity were analyzed in model cells for liver using human hepatoma cells (HepG2; from ATCC) and mammary epithelial cells (MEC) using the bovine mammary epithelia cell line MAC-T (kindly provided by Prof. U. Dobrindt, University of Münster, Germany). HepG2 cells were grown in MEM Earle’s medium (Biochrom, F0315) supplemented with 10 % (v/v) heat-inactivated fetal calf serum (FCS), 1.5 g/L sodium bicarbonate, 2 mmol/L l-Glutamine, 0.1 mmol/L non-essential amino acids and 1 mmol/L sodium pyruvate. MAC-T cells were cultured in Dulbecco’s modified Eagle’s medium (Lonza, B-4800) containing 5 % (v/v) heat-inactivated FCS and 4 mmol/L l-Glutamine.

All plasmid DNA used for transfection was prepared free of endotoxin using the NucleoBond Plasmid Maxi Xtra Purification kit (Clonetech). For transient transfections, cells were split the day before into 6-well plates and seeded in medium omitting antibiotics. Plasmids containing the luciferase reporter gene and the respective expression constructs were co-transfected using Lipofectamine 2000 (Life Technologies, Inc), essentially as previously described in detail [51]. The amount of transfected DNA was kept constant, especially in dose finding experiments by eventually filling up to a constant amount with enough DNA of the empty vector of the expression constructs (pcDNA3.1+). The lipofectiamine and plasmid DNA containing transfection mixture was replaced after 3 h with the respective growth medium. Cells were lysed after 48 h with the passive lysis buffer (Promega) and the luciferase activity was measured using the dual luciferase assay reporter system (Promega). Luciferase activity was measured with a Berthold luminometer and normalized relative to the protein content of the sample. The latter was determined with the BioRad-protein-assay kit.

### Rapid amplification of 5′-cDNA ends (RACE)-PCR

Primers for RACE-experiments are listed in Additional file 1: Table S1B. 5′ RACE experiments used the GeneRacer™ kit (Invitrogen) essentially as prescribed. Total RNA extracted from healthy liver tissue served as template to synthesizing cDNA with the specific reverse primer (Rpr2) and oligo dT using the Superscript II reverse transcriptase (invitrogen). Purified cDNA (High Pure PCR Purification Kit; Roche) was amplified in primary and nested PCR reactions with the primer pairs Rpf2/Rpr2 and Rpf1/Rpf1, respectively. The heaviest DNA band of the PCR products was retrieved and cloned into pGEM-T Easy (promega) and sequenced. The 5′ most located transcription start site was determined by comparison with the bovine genomic sequence as given in file AY241932.

### In silico analysis of transcription factor binding sites on the promoter

The DNA-sequence analysis of the SCD-encoding gene was based on file AY241932. Potential binding sites for transcription factors were searched for with the MatInspector program (http://www.genomatix.de/matinspector.html). Filters were set ≥0.95 for similarity of the core sequence and ≥0.88 for the surrounding sequence.

### Statistical analysis

Significance of different mean values found between groups was evaluated in an unpaired *T* test as provided in the Microsoft excel program package. P < 0.05 indicated a significant difference.

## Results

We exploited for our study as in vivo model tissue samples (udder, liver) having been collected during a well-controlled *E. coli* mastitis infection trial of first lactating Holstein Friesian heifers. The trial included a comparison with age and lactation stage matched entirely healthy cows, serving as “gold standard” controls [6].

### *E. coli* mastitis reduced SCD1 expression in liver and udder

We first validated with RT-qPCR measurements that the expression of SCD1 was indeed reduced in liver and infected udder quarters of our infected cows. The SCD1 mRNA concentration in livers from the mastitis group (3902 ± 1909 [relative cDNA copy number, ± S.E.M.]) was only 1/3 of that found in age and lactation stage matched healthy cows (10,419 ± 1832; Table 1). The relative SCD1 mRNA concentration was reduced in infected udder quarters; from 203,689 ± 30,739 (mean ± S.E.M.) cDNA copies generated from healthy glands down to only 1/5 of this concentration in samples generated from infected quarters (40,405 ± 3024). It was also reduced by approximately 30 % in the sterile udder quarters neighboring the infected ones. We determined the concentration of the ACACA-encoding mRNA as a control parameter for the overall rate of fatty acid synthesis. This was unchanged in livers from infected and healthy cows, but 2-fold down regulated in the infected udder quarters.

**Table 1:** SCD1 and ACACA expression in tissues from experimental animals

Condition	MG		Liver	
	SCD1	ACACA	SCD1	ACACA
Healthy	203.69 ± 3.07	11.46 ± 1.50	10.42 ± 1.83	1.83 ± 0.34
Infected	40.41 ± 3.02^**^	5.19 ± 0.23^**^	3.90 ± 1.91^*^	2.08 ± 0.61
Neighbor quarter	138.32 ± 19.84^**^	10.08 ± 1.52	–	–

Mean (± SEM) cDNA copy numbers (x10^3^); liver: n, 6 (healthy); n, 5 (infected); MG (udder): n, 5 for each group

* P < 0.05; ** P < 0.01

### Definition of the SCD1 promoter and putative transcription factor binding sites

The promoter expressing the SCD1-encoding gene in cattle has previously not experimentally been defined. Rather, it was assumed that it would similarly be positioned as in the homologous genes from mouse and human [52]. We therefore identified the location of the tsp in 5′-RACE experiments using liver RNA from our control animals as template. We define as tsp the position found in the most 5′-reaching isolate from among 14 clones having been sequenced (Fig. 1a; Additional file 2: Figure S1). Another four clones revealed position +3 as 5′-end, while the transcripts identified by six more clones initiated at position +6. Our newly defined tsp at position +1 resides 73 upstream of the previously presumed location, 227 bp upstream of the start codon of translation. The latter position is widely used as a reference point in studies dealing with the SCD1 promoter.

The in silico analysis of the promoter sequence revealed attachment sites for several potentially relevant modulators of transcription and transcription factors (Fig. 1a; Additional file 2: Figure S1). These included two factors known to modulate chromatin compaction (SWI/SNF; RBP2; [53, 54]) but also key activators of SCD1 expression (SREBP1, C/EBP) and other highly relevant regulators of fatty acid synthesis (PPAR; NF-Y; AP2, SP1). These have all been previously recognized in other studies. We found in addition previously unrecognized attachment sites for NF-κB, those mediators of inflammation related reprogramming of cellular metabolism. Intriguingly, the two NF-κB binding sites are closely adjacent and each site is almost overlapping with a C/EBP factor binding site.

### Mastitis did not alter the chromatin structure of the SCD1 promoter in liver

In searching for mechanisms downregulating SCD1 expression in the target tissues, we examined first if infection would result in tightening the chromatin particularly around those putative binding sites for enhancers of SCD1 expression. Operation of such epigenetic mechanisms seemed plausible since we have previously shown in the very same liver samples that infection had loosened the chromatin in promoters of key immune genes [45]. However, measuring in the current study from the same samples with the same technique the degree of chromatin compaction in three different areas (cf Fig. 1a, areas A, B, C) of the SCD1 promoter did not reveal any infection related changes in liver samples (Table 2). Also, there was no correlation between the degree of chromatin compaction in any of the three areas and the degree of SCD1 expression, as indicated by the mRNA concentration (Fig. 1b). Hence, we found no evidence that epigenetic mechanisms acting through chromatin remodeling might significantly contribute to downregulate SCD1 expression in liver during acute mastitis.

**Table 2:** Degree of chromatin compaction (%) of the SCD1 promoter in livers

Area	GS	Infected
A	61 ± 4	55 ± 4
B	50 ± 6	50 ± 3
C	65 ± 9	57 ± 5

See map in Fig. 1 for area location; values are mean ± S.E.M. from n = 6 (GS, healthy) or n = 5 (infected) samples. For each sample the mean value from two separate determinations was used

### Infection differentially modulated the expression of SREBP1a and C/EBP factors in udder and liver

**Fig. 2:**
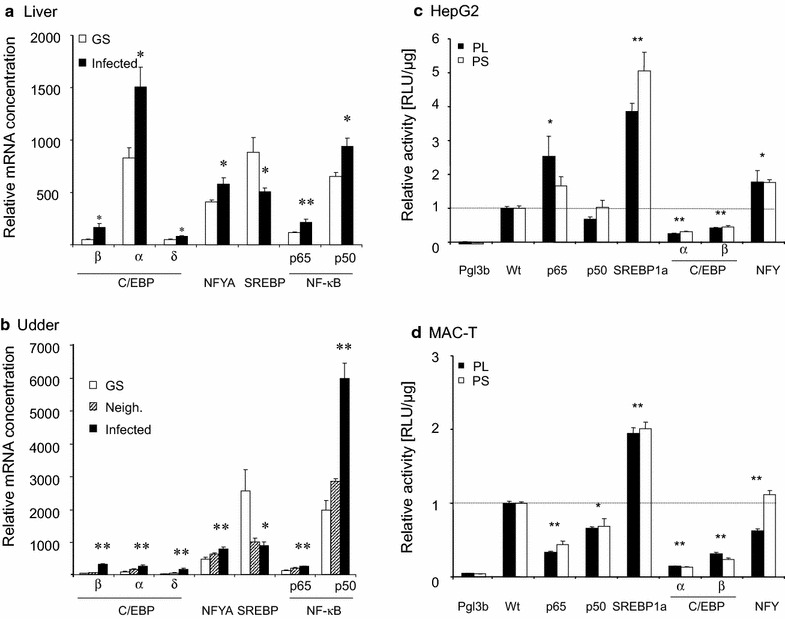
Expression of transcription factors (TF) in livers (**a**) or udders (**b**) and effect of overexpressing TFs on the promoter of SCD1 in HepG2 (**c**) or MAC-T (**d**) cells. The concentration of the mRNAs encoding the respective factors was determined in RT-qPCR and was normalized against 1000 copies of the SFRS4 factor. **a** Liver samples were from healthy (GS) or udder infected cows (infected). **b** Udder samples were from healthy cows, or from sterile quarters neighboring (Neigh.) infected (Infected) ones. *Error bars* represent S.E.M.; *asterisks* indicate significance of difference to the value from healthy control cows (*P < 0.05; **P < 0.01). Reporter gene constructs (1 µg) expressing the Firefly-luciferase under the control of the long (PL) or short (PS) promoter segment were transfected into the **c** HepG2 or **d** MAC-T cells, together with 0.2 µg of vectors expressing the bovine transcription factors, as indicated. Lucifearse activity was assayed 48 h later from triplicate wells. Activity was calculated as RLU/µg of protein lysate. Represented are the mean values from two independent determinations and expressed as fold changes relative to controls set as 1.0 (*dotted line*), having received the reporter gene together with the empty vector used for establishing the expression constructs. Control measurements of the promoter-less pGL3-basic vector are also shown (Pgl3b). *Error bars* represent S.E.M.; *asterisks* indicate significance of difference to the value from healthy control cows (*P < 0.05; **P < 0.01)

We next evaluated which of the putative transcription factors might be involved in the infection caused downregulation of SCD1 expression. We used the infection related changes in the mRNA abundance of the respective factors as indicator for their potential functional involvement. All candidate factors revealed some infection related changes in their mRNA abundance (Fig. 2a, b; Additional file 1: Table S3A lists the respective data). Levels of the SREBP1a-encoding mRNA were significantly down regulated, by 1.8-fold in liver and almost 3-fold in the infected udder quarters. The sterile udder quarters neighboring the infected ones had SREBP1 mRNA levels almost as low as the infected quarters. This indicates their regulation through systemic rather than local factors. Infection had significantly increased the mRNA concentrations of our other candidate factors over the levels found in the control samples. Of note, also concentrations of the mRNAs encoding the C/EBP factor series (α, β, δ) were all significantly higher in the liver samples from the infected animals or infected udder quarters.

### SREBP1a activated while C/EBP factors downregulated the activity of the SCD1 promoter

We established two reporter gene constructs to enable molecular analyses of the role of those candidate factors for regulating SCD1 expression. Therefore, a long (1767 bp; Fig. 1a) and a distally truncated (884 bp) segment of the SCD1 promoter were used to drive the expression of a luciferase expressing reporter gene. These constructs were transfected (1 µg) into HepG2 or MAC-T cells, serving as models for liver and mammary epithelial cells, respectively. For a first survey of a possible regulatory role of our candidate transcription factors, we co-transfected either of the two reporter gene construct together with 200 ng of different vectors expressing either of the factors NF-κBp65; −p50; SREBP1a; C/EBPα; −β and an equimolar mixture of vectors expressing the factors NFY A, -B, -C. NFY and NF-κBp65 stimulated the promoter activity in HepG2 cells, while NF-κB p50 was neutral (Fig. 2c). Modulations of the promoter activity through NF-κB factors must have reflected off-target effects, since the short promoter does not feature a NF-κB binding site. These factors were all neutral or slightly repressive in MAC-T cells (Fig. 2d). However, we found as consistent effect in both model cells that SREBP1a strongly and significantly activated the promoter (approximately 2- to 5-fold over the controls), whereas expression of either of the two C/EBP factors −α or −β significantly quenched the promoter activity, down to 50 % or less.

**Fig. 3:**
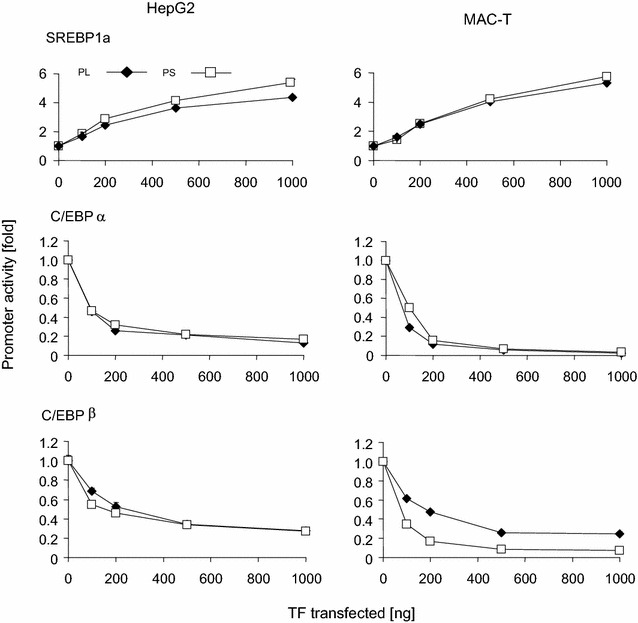
Response of promoter activity to increasing dose of SREBP1a or C/EBPα or −β transcription factors in HepG2 or MAC-T cells. Reporter genes (1 µg) with the long or short promoter segment (PL, PS) were transfected into HepG2 or MAC-T cells, together with increasing amounts (abscissa) of vectors expressing the respective transcription factor. RLUs (ordinate, untreated control set as 1) were determined 48 h after transfection, as described above. The total amount of transfected DNA was kept constant by eventually filling up with empty vector. Values are means calculated from two independent determinations, each assayed in triplicate. S.E.M. *error bars* of the mean values were always smaller than the size of the symbols. All, but the values for 100 ng of transfected SREBP were significantly different from the controls

We scrutinized the roles of those factors exhibiting consistent effects in both model cells, since functional consistency clearly indicated their high relevance for regulating the SCD1 promoter activity. Transfecting increasing amounts of these expression vectors revealed in all cases the dose dependence of the effect (Fig. 3). SREBP1a transfection similarly increased the activity of both promoter segments, eventually by more than 4-fold in both model cells. In contrast, both C/EBP-factors quenched the promoter activity very clearly in a dose dependent fashion. C/EBPα reduced the promoter activity of both promoter constructs in HepG2 cells to less than 20 % (Fig. 3). The repressive effect was even stronger in MAC-T cells. Here, high amounts of transfected expression vector (1000 ng) reduced the residual activity of both promoters to only less than 5 % of the control.

Only in HepG2 cells appeared the effect of transfected C/EBPβ upon promoter activity to be similar as that of C/EBPα. The residual activity of the long and short promoter segments was eventually lowered to only 27 or 28 %, respectively (Fig. 3). In MAC-T cells, however C/EBPβ quenched the activity of the short promoter segment to a larger extent than that of the long one. The residual activity of the long promoter segment would remain at ~25 % even if high amounts of the C/EBPβ vector were transfected but that of the short promoter was quenched down to almost the same low values as recorded from C/EBPα (< 5%).

### C/EBP binding sites were repressive in HepG2, but not in MAC-T cells

Given that C/EBP factors have so far been known to activate rather than repress SCD1 expression we analyzed in more detail the mechanism of C/EBP factor mediated repression in both of our models cells. We therefore mutated two from among the three putative C/EBP binding sites (see map in Fig. 1a) in order to evaluate their significance for promoter activity. On the long promoter construct, we mutated the distal site either alone or together with the proximal site. The proximal site was mutated on the short promoter segment. These constructs were transfected into both model cells (HepG2, MAC-T) and their activity was directly compared to those of the respective wt-promoter variants.

**Fig. 4:**
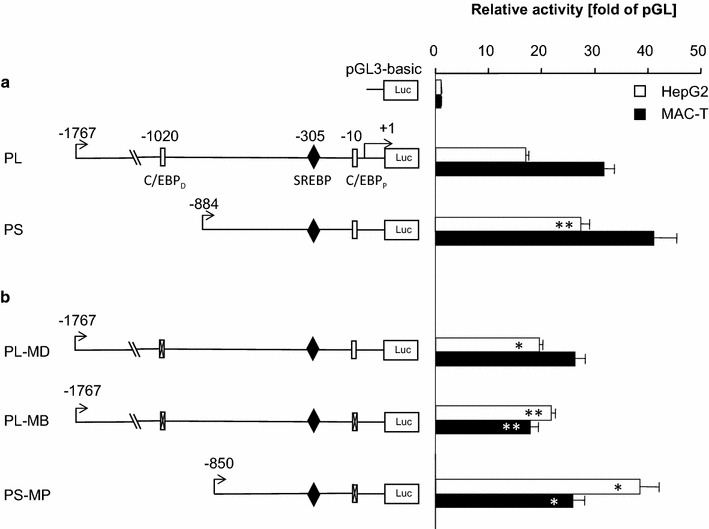
Contribution of C/EBP binding sites on the basal promoter activity. **a** Reporter genes harboring the long (PL) or short promoter (PS) segment were transfected into HepG2 or MAC-T cells. The relative luciferase activity was determined 48 h later and was expressed as multiple of the activity of the promoter-less vector pGLbasic (abscissa). Position of the distal and proximal C/EBP binding sites is indicated (C/EBP_D_; C/EBP_P_). **b** Activity of reporter genes in which either the distal C/EBP binding site (at −1020; PL-MD) or both sites were mutated (PL-MB). In addition, the proximal binding site (at −10) was mutated on the short promoter alone and the effect was recorded using the construct PS-MP. Values are the means from two experiments, each assayed in triplicate. *Asterisks* indicate the statistical significance of the difference towards the construct with the respective wt-promoter

Considering first the effect of truncation of the wt-promoter on the basal promoter activity, we found that deletion of the distal segment slightly, but significantly enhanced promoter activity in HepG2 cells (+60 %; Fig. 4a). The effect was in tendency similar in MAC-T cells (+30 %; P, 0.08).

All 3 mutations slightly enhanced the promoter activity in HepG2 cells, by 14, 28 and 41 % for the distal, double or proximal mutations, respectively (Fig. 4b). In contrast, all three mutations lowered the promoter activity in MAC-T cells, down to 83, 56 and 82 % by the distal, double or proximal mutations. The data consistently revealed an opposite effect of the C/EBP binding sites in both cell types. They slightly enhanced the promoter activity in HepG2 cells but lowered it in MAC-T cells.

### C/EBPα, but not C/EBPβ exerted its effect in cis

The mutation analyses of the significance of the C/EBP binding sites had revealed only slight and even controversial effects in both cell types. Hence, we wanted to characterize in more detail the molecular mechanism by which these factors repress the SCD1 promoter. We focused on the factors C/EBPα and C/EBPβ, given the well-known significance of the α-factor for adipogenesis and the important role of the β-factor immune functions and lipid synthesis in MEC. We applied two complementary approaches. On one hand, we examined the effect of the full length transcription factors on the reporter genes harboring the mutated promoters. On the other hand we titrated on the wt-promoters the effect of truncated versions of the C/EBP factors in which the transactivation and repressor domains had been deleted. Such engineered factors are known as trans dominant negative (‘DN−‘) factors. Both activator domains and the attenuator (repressive) domain had been deleted in the DN-C/EBPα factor. Both activator domains had also been deleted in the DN-β factor together with the N-terminally positioned of the two repressor domains. However, the second repressor domain was retained in DN-C/EBPβ (see schematic representation of the factor domains in Additional file 2: Figure S2).

**Fig. 5:**
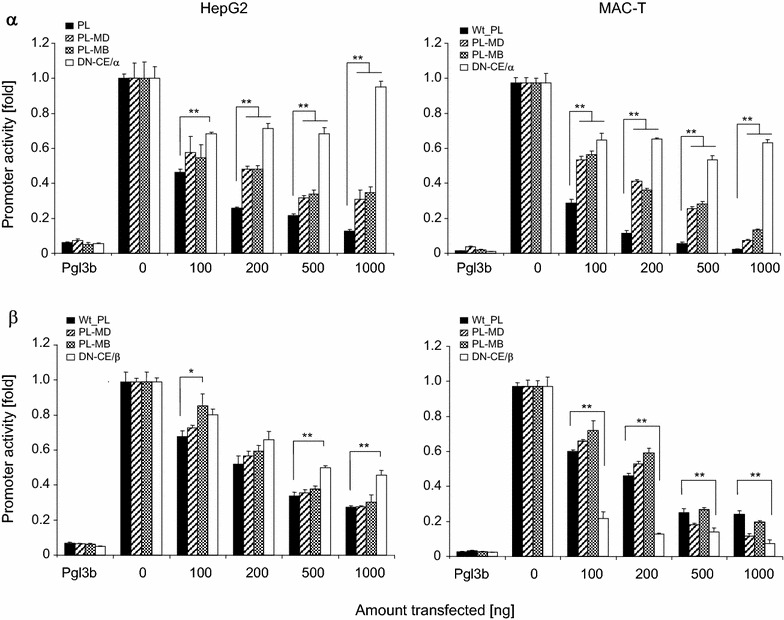
Differentiation of the effect of C/EBPα and −β on the promoter activity. Increasing amounts of vectors (100–1000 ng) expressing C/EBPα (α, *upper panels*) or −β (β, *lower panels*) were transfected into HepG2 or MAC-T cultures harboring the long segment of the wt-promoter (*black columns*) or its variants with either the distal (*striped columns*) or both C/EBP binding sites mutated (*checkerboard texture columns*). Luciferase activity was recorded 48 h later and expressed as fraction of the control cultures with no C/EBP expression vector added (0 ng) set as 1. These assays were compared to the effect of increasing amounts of trans-dominant negative variants (DN-CEBP) of the respective C/EBP factors on the wt-promoter (*open columns*). Values are means from two experiments each assayed in triplicate. *Asterisks* indicate statistical significance of difference (*P < 0.05; **P < 0.01)

Regarding the effect of the full length C/EBPα factor, we found that it functioned consistently in both cell lines in a dose dependent fashion. Increasing amounts of this factor were less repressive on both mutated promoters than on the wt-promoters (long promoter, Fig. 5; short promoter, Additional file 2: Figure S3). This difference was statistically significant (P < 0.05) after transfecting 200 ng or higher amounts of the factor. The effects were clearest at the high dose (1000 ng) of the transfected expression construct. This concentration lowered the activity of the wt-promoter in the HepG2 cells down to only 13 %, but the residual activity of the constructs featuring the distal or doubly mutated C/EBP sites was still 37 and 39 % respectively (Fig. 5 upper left panel). This difference indicated a C/EBP binding site dependent approximately 3-fold reduction of the repression through C/EBPα. The clear difference between the wt vs mutated C/EBP-binding site upon C/EBPα factor mediated regulation of the SCD1 promoter activity validated by inference that the wt -promoter does indeed bind the C/EBP factor.

Transfection of the DN-C/EBPα factor confirmed that the repressor function of this factor resides in those domains know to directly modulate transcription. Transfecting increasing amounts of the DN-C/EBPα expressing vector into HepG2 cells quenched the promoter activity to some extent. However, the effect was much weaker than exerted by the full-length factor and was not dose dependent. Even at the highest dose having been transfected (1000 ng) was the activity of the reporter almost as high measured from the controls having not received any C/EBP factor expressing vector.

We obtained in MAC-T cells similar results as in HepG2 cells concerning the effects of the full length- or the DN-C/EBPα factor, using both the wt-type and the C/EBP binding site mutated promoter variants (Fig. 5 upper right panel). Effects were even clearer using the short promoter as driver for the reporter gene (Additional file 2: Figure S3). The data all together show that the C/EBPα factor indeed acts as a repressor on the SCD1 promoter in a DNA-binding sequence motif and factor domain dependent fashion.

Results were different and less clear when using the C/EBPβ factor in identical experimental settings. Considering first the effects in HepG2 cells, we observed neither for the long nor the short promoter any significant difference between wt-type and mutated promoters regarding the dose dependent repression efficacy of the full length C/EBPβ factor (Fig. 5, lower left panel; Additional file 2: Figure S3). Transfection of 1000 ng of the expression construct lowered the residual activity of wt and mutated promoters down to 28 % for wt and the single mutated promoter; and 31 % for the doubly mutated promoter. Hence, repression of SCD1 promoter activity through C/EBPβ was not dependent on binding to the DNA-sequence in HepG2 cells.

Transfecting the DN-C/EBPβ factor also did not as clearly as DN-C/EBPα reveal a reduced repressor efficacy compared to the full length factor. However, transfecting high concentrations (500, 1000 ng/well) of the vector expressing this factor into HepG2 cells revealed a slight and statistically significant reduced repression efficacy compared to the full length factor (Fig. 5 lower left panel). This effect was even stronger and very obvious when using the short promoter (Additional file 2: Figure S3). Hence, C/EBPβ dependent repression of the SCD1 promoter clearly involved in HepG2 cells the N-terminal part of the factor comprising the activation domains and the repressive domain 1 (RD1).

In MAC-T cells, however we neither found any DNA-binding sequence motif nor a factor domain dependent direct repressive effect of C/EBPβ. The full length factor was as repressive for the activity of the wt-promoters as for the mutated promoters (Fig. 5, lower right panel). Moreover, the truncated DN-C/EBPβ variant appeared to be an even stronger inhibitor for the activity of the long promoter than the full length C/EBPβ factor.

Our data together show that the molecular mechanisms by which C/EBPβ represses the SCD1 activity are different from those of C/EBPα. Repression through C/EBPβ is apparently independent from the DNA-sequence binding motif and does not involve the activator or attenuator domains. Moreover, there is a cell type specific modulation of the mechanisms by which C/EBPβ exerts its repressive function on the SCD1 promoter.

### SREBP1a and C/EBPα independently exert their effect on the SCD1 promoter

**Fig. 6:**
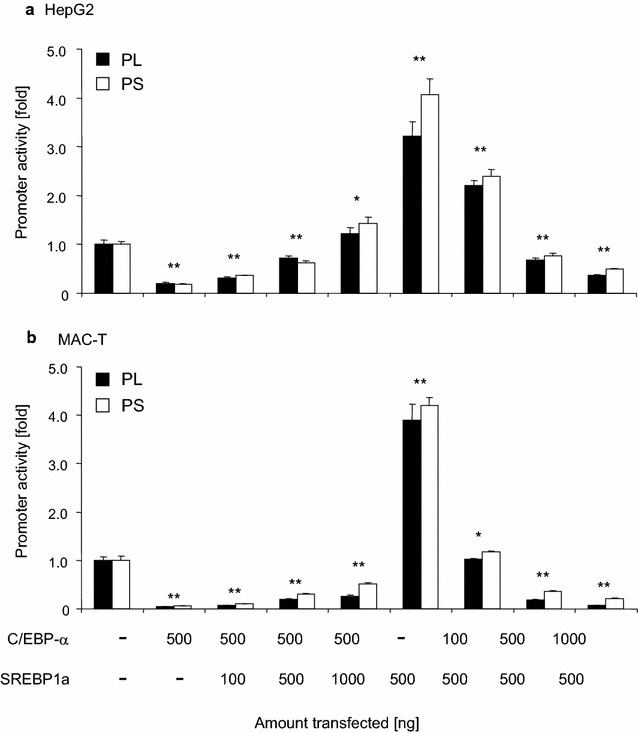
SREBP1a and C/EBPα exert their effects independently from each other. Reporter genes (1 µg) with the long or short promoter segment (PL, PS) were transfected into HepG2 or MAC-T cells together with 1000 ng of empty vector (−), 500 ng expressing C/EBPα or SREBP1a. Increasing amounts of vectors expressing either SREBP1a or C/EBPα (*cf* legend below figure) have in addition been co-transfected in other dishes. The total amount of transfected DNA was kept constant in all assays by eventually filling up with empty vector. RLUs/µg of protein were determined 48 h after transfection and normalized against the control, set as 1. Values are means from two experiments, each assayed in triplicate. *Asterisks* indicate statistical significance of difference relative to the control having not received any expression vector for transcription factors (*P < 0.05; **P < 0.001)

We wondered if either the enhancer SREBP1a or the repressor C/EBPα would override the effect of the respective other factor. We therefore co-transfected a fixed amount (500 ng) of either the C/EBPα or the SREBP1 expression vector together with the reporter gene construct harboring the long promoter. Some of the C/EBPα transfections received in addition increasing amounts (100–1000 ng) of the SREBP1 expression vector. Similarly, some of the SREBP1 transfections were supplemented with increasing amounts (100–1000 ng) of the C/EBPα expression vector. We found in both model cells that increasing amounts of the SREBP1 expression vector would significantly relief the repression through C/EBPα (Fig. 6); and that increasing amounts of C/EBPα would reduce the stimulatory effect exerted by the SREBP1 expression construct. We noted as cell type specific difference that the repression through C/EBPα was stronger in MAC-T than in HepG2 cells, similar as noted in the previous experiments (cf Fig. 3).

## Discussion

Key motivation for our study was to see if epigenetic mechanisms would play a significant role in downregulating SCD1 expression in liver during metabolic disorders caused through increased levels of circulating LPS. If true, this would have opened novel strategies for the veterinarian to intervene systemically against the detrimental metabolic effects of acute mastitis or SARA, for example by using small molecular inhibitors of histone modifiers (see [55] for a review). The samples serving as in vivo model for the current study had been collected during a previous trial into experimentally elicited *E. coli* mastitis. Clinical analyses and global transcriptome profiling had proven that the udder infected cows had suffered from clinical mastitis which had been accompanied by a strong systemic reaction [6]. All the infected udders had been reprogramed from lactation to immune defence, including a shutdown of milk and milk fat synthesis, similar as previously reported [4]. Livers of the very same cows had also revealed that chromatin remodeling had contributed to upregulate the expression of immune genes including those contributing to the acute phase response [45].

We made two novel key observations regarding the down-regulation of SCD1 expression. First, no evidence was found for infection related enforced chromatin compaction of the SCD1 promoter in the livers of the experimental cows. Second, we found—in contrary to the widespread opinion in literature—that the C/EBP factors −α and −β are repressors rather than activators of SCD1 expression in liver and mammary epithelial cells.

### No evidence for involvement of chromatin remodeling or NF-κB factors in downregulating SCD1 expression during inflammation

For detecting signs of the involvement of epigenetic mechanisms we monitored in liver samples the degree of promoter compaction over an area exceeding 1000 bp and covered also that proximal area at around that SREBP1a/NFY binding site (area B, Fig. 1a; Additional file 2: Figure S1) which had been identified in numerous studies (including cattle) as key driver element of SCD1 expression [29–31]. In neither of the areas did we detect any signs of infection related changes in chromatin compaction. The technical validity of our observation at the SCD1 promoter is underscored by the fact that we have previously reported infection related chromatin de-compaction at the promoters of immune genes using the very same samples and techniques [45]. Those regulated genes had included TLR2, -4, LBP, HP. The chromatin loosening of their promoters had been contrasted by revealing the unaltered chromatin compaction of the αS1-casein promoter. Moreover, mastitis-related hypomethylation of the TLR4 promoter had also been found in these samples. On the background of these controls it is clear that our current study did not yield support for the concept to eventually counteract LPS-related disturbances of fat metabolism in liver through intervening systemically with modulators of epigenetic mechanisms. Sample limitation prohibited to also examine chromatin from udder tissue of the same animals.

We therefore set out to look for alternative mechanisms downregulating SCD1 expression and conducted reporter gene assays in well-established model cells for MEC [56, 57] and liver cells [58]. Overexpressing the SREBP1a factor validated the well-known crucial role of this factor for stimulation of SCD1 expression [30]. The results obtained with vectors expressing the factor series NFY-A, -B, -C are also in line with the respective literature in that we observed in the HepG2 model for liver cells a significant stimulation (cf [24]) and slight repression in the MAC-T model for MEC, similar as recently reported using the human breast cancer line MCF7 [59]. The pivotal role of the NFY factor family for the basal and nutritional regulated activity of the SCD1 promoter has recently been underscored in goat MEC model cells [60]. We had included into this set of experiments vectors expressing the NF-κB p50 and −p65 factors not least because of the close vicinity of their cognate promoter binding site to each other and that of C/EBP factors. Closely spaced—or even overlapping—binding sites for these two classes of transcription factors have been conserved throughout vertebrate evolution on promoters of immune relevant genes ([61] and references herein) and their interplay may regulate expression of immune genes in a gene- and tissue-specific fashion [39]. However, overexpressing NF-κB p50 was almost without any effect for the SCD1 promoter activity and overexpression of NF-κB p65 exhibited opposite effects in MAC-T and HepG2 cells. Moreover, co-expressing either of the NF-κB factors with C/EBPα did not reveal any signs for factor co-operation (data not shown). Hence, modulated expression or activity of NF-κB factors cannot be the key to downregulate during inflammation the SCD1 expression commonly in udder and liver.

### C/EBP-α and −β are repressors in MEC and liver cells and the −α factor acts in cis

The C/EBP factors −α and −β both regulated SCD1 promoter activity into the same direction in both model cells, which was—surprisingly enough—downward! C/EBP factors have never before been reported as negative regulators of SCD1 expression, to the best of our knowledge. Yet, expression constructs for both of these factors strongly quenched the activity of the SCD1 promoter in a dose dependent fashion in both model cells. Proper expression and DNA-sequence dependent binding of these factors and their DN-variants has previously been documented [39, 40, 50]. Our data regarding the role of C/EBPα as a repressor for the activity SCD1 in MEC and liver cells are consistent in all aspects and in both of our model cells. This factor repressed SCD1 activity in a DNA-sequence motif and repressor domain dependent fashion, leaving no doubt that it properly exerted this function acting directly in cis.

The role of C/EBPα is different in adipocytes. Christy et al. [27] expressed C/EBPα in 3T3-L1 preadipocytes (from mouse) and convincingly proved strong activation (>20-fold) of the murine SCD1 promoter through this factor. Others, however only inferred a C/EBP factor dependent activation of the SCD1 promoter just from the observed unidirectional and co-regulated expression of SREBP1 and C/EBP factors in the same cells [28, 32] or the presence of C/EBP factor DNA-binding motifs on the promoter [30]. However, C/EBP factors exert multiple functions and their expression is under multifactorial controls [62]. Combining our data with the evidence from literature suggests a tissue specific differentiated function of C/EBPα for regulating SCD1 expression, acting repressive in liver and mammary epithelial cells but as an enhancer in adipocytes. Recognizing tissue specific adverse functioning of transcription factors is not uncommon. We have previously observed, for example that NF-κBp65 may either enhance or strongly repress the activity of the CXCL8 promoter from cattle depending upon the cell-type [39].

### C/EBPβ is known to sometimes acting in trans

The efficacy of C/EBPβ to repressing the activity of the SCD1 promoter in both of our model cells was similar as that of C/EBPα but apparently mediated through a different mechanism. Repression of the β-factor was independent from the DNA-sequence motif. This requires assuming that the effect of C/EBPβ is relayed onto the SCD1 promoter through interaction with a different factor. C/EBP factors (both, −α and −β) are long-known to eventually heteromerize in particular with NF-κB p50 [41, 43, 44]. The NF-κB p50 factor features no trans-activation domain. However, its heteromers with C/EBP factors may eventually exploit the NF-κB p50 factor binding to its cognate site at the promoter and use the trans-activation domain of the C/EBP factor to stimulating activity of the target promoter [63]. It was found in a reciprocal observation that the Jun-factor repressed the activity of the surfactant-associated protein B promoter only if being tethered to C/EBP-factors (either −α or −β) having bound to their cognate site at the promoter [64].

Clearly, our experimental settings and data do not allow identifying the nature of such a factor possibly interacting with C/EBPβ. However, the suggested interaction of C/EBPβ with an auxiliary factor in MEC to corporately regulate the SCD1 promoter might bear clues for understanding the apparently cell-type specific differentiated effect of C/EBPβ on the activity of the SCD1 promoter. Moreover, such a multi-layered mode of action could also help to understand, why this factor—acting in cis—is a strong enhancer of ACACA expression in MEC [40], while repressing the SCD1 promoter in the same cell type.

## Conclusions

Our study shows that downregulation of SCD1 expression in liver during LPS-caused metabolic disorders does not involve chromatin remodeling at the promoter. Rather, concerted down-regulating the expression of the enhancer SREBP1 with concomitantly enforcing expression of the repressors C/EBPα and −β readily explains the downregulated SCD1 expression occurring locally in the udder and distantly in liver during the systemic reaction elicited by acute mastitis. This explanation rests on our solid prove that the C/EBP factors are repressors of SCD1 expression in liver and mammary epithelial cells (MEC). Comparing our respective data with literature suggests that C/EBPα exerts a cell type dependent function on the SCD1 promoter, acting as an enhancer in adipocytes but as a repressor in hepatocytes and MEC.

## Additional files

10.1186/s12867-016-0069-5 Stability of the expression levels of selected reference genes. RT-qPCR determination of the mRNA concentration of SFRS4, ACTB, CLIC1, RPLA35 in livers and udder samples from healthy or infected cows. **Table S2.** List of primers used for RT-qPCR (S2A) and vector constructions (S2B) and of the respective DNA-sequence source files. **Table S3.** Relative copy numbers of mRNAs encoding transcription factors in tissues (S3A) and model cells (S3B).
10.1186/s12867-016-0069-5 DNA sequence comparison of the SCD1 promoter from cattle and human indicating positions and motifs of relevant transcription factors and restriction sites. **Figure S2.** Map of the domains of wt and DN-variants of the factors C/EBPα and −β. **Figure S3.** Differentiation of the effect of C/EBPα and −β on the activity of the truncated short promoter. Data were achieved in identical experimental settings as shown in Fig. 5, however using the reporter gene construct PS.

## Abbreviations

ACACA: acetyl-CoA carboxylase-alpha
C/EBP: CCAAT enhancer binding protein
LPS: lipopolysaccharide
NF-κB: nuclear factor kappa-B
NFY: nuclear factor Y
RACE: rapid amplification of cDNA ends
SREBP1: sterol regulatory element binding protein 1
Tsp: transcriptional start point
