# Supplementary material for: Stearoyl-CoA desaturase 1 expression is downregulated in liver and udder during E. coli mastitis through enhanced expression of repressive C/EBP factors and reduced expression of the inducer SREBP1A
Source: BMC Mol Biol. 2016 Jul 20;17:16. doi: 10.1186/s12867-016-0069-5 (PMC4955114; doi:10.1186/s12867-016-0069-5)
Supplement: Supplementary file 1 — 10.1186/s12867-016-0069-5 Stability of the expression levels of selected reference genes. RT-qPCR determination of the mRNA concentration of SFRS4, ACTB, CLIC1, RPLA35 in livers and udder samples from healthy or infected cows. Table S2. List of primers used for RT-qPCR (S2A) and vector constructions (S2B) and of the respective DNA-sequence source files. Table S3. Relative copy numbers of mRNAs encoding transcription factors in tissues (S3A) and model cells (S3B). [file 12867_2016_69_MOESM1_ESM.pdf]

Additional file 1, Supplementary table S1**:** Stability of the expression levels of reference genes

Tissue	Gene	GS	Infected	Fold[a]	p[b]
Liver	*SFRS4*	9991 ± 435	9174 ± 763	0.92	0.40
	ACTB	240189 ± 14225	413546 ± 61026	1.72	0.055
	CLIC1	5029 ± 579	8788 ± 1022	1.75	0.02
	RPLA35A	250703 ± 3446	425551 ± 61529	1.70	0.03
Udder	*SFRS4*	7143 ± 922	9591 ± 632	1.34	0.06
	ACTB	45225 ± 1775	150757 ± 15931	3.33	0.00
	CLIC1	11184 ± 2106	128123 ± 16900	11.4	0.00

Values are mean values (± S.E.M.) of copy numbers, relative to the same amount of cDNA input in every sample. GS (Gold standard), n=6; Infected (*E. coli*, 24 h), n=5; [a]Fold, fold change of mean values (infected vs GS samples); [b]P value of difference (Student's t-test).

Additional file 1; Supplementary Table S2A: Primers for real time quantitative PCR.

Gene	Accession#	cDNA generation (5'-)	Forward primer (5'-)	Reverse primer (5'-)	Amplicon (bp)
SCD1	NM_173959.4	GCAGCAAGTGGGCCGGCAT	CACGCAAAAGCAGGCTCAGGA	GCATCTGGGCTCTCAGACACT	110
ACACA	NM_174224.2	TTTCAAGAGAAGTTCTGGGAGCT	GTTCTCATTGCCAACAATGGCA	CAGCCAGCCCAAACTGCTTGCAC	266
C/EBPα	NM_176784.2	GCAGTTGCCCATGGCCTTG	GCAAGAGCCGGGACAAGG	TCGGGCAGCTGACGGAAG	151
C/EBPβ	NM_176788.1	5'-TTTTTTTTTTTTTTTTTTTTN	GACAAGCACAGCGACGAGTACA	CGACAGTTGCTCCACCTTCTTC	198
C/EBPδ	NM_174267.2	5'-TTTTTTTTTTTTTTTTTTTTN	AACCCGCTGCCTTCTACGA	CCCGCTTTGTGGTTGCTGT	152
NFYA	NM_001014956.1	5'-TTTTTTTTTTTTTTTTTTTTN	AGGCGGACGATTTTTCTCT	AGTGGGAAACTGTGAGGAAT	150
SREBP1a	NM_001113302.1	CAGCTTCTGCCGCTGCCAGA	GCCAGCCTGGCAATGTGTGA	CAGGTCACACAGGAGCAGCT	103
NF-κB (p65)	NM_001080242.2	AAGTTGATGGTGCTGAGAGATG	TTTTTCACAAGCTGACGTGCAC	GCTCTTGAAGGTCTCGTACGT	211
NF-κB (p50)	NM_001076409.1	CACTGTCCCCGTTCTCATC	GTCAAACTCCAGAATGGCAGA	GAAATCCTCTCTGTTTAGGTTGCTC	179
SFRS4	NM_001101231.1	CTTGTGGGCATCTGCATAAGTC	ATGGCAGTTACGGTTCTGGAC	CCTGCCTGACGCATATAATCC	156

The source files used to derive the primers are indicated (accession#).

Additional file 1; Supplementary Table S2B: Primers for plasmid constructs, chromatin assay and 5’RACE experiment.

Primer name	Sequence (5'-3')*,#	Applications
pf1	AGCTGGTACCGGAGCAACACCTGCCCAGAC	WT promoter construct (PL)
pf2	AGCTGGTACCGTGACCATTGGCTGAACCTCT	WT promoter construct (PS)
pr2	AGCTAAGCTTGCATCTGGGCTCTCAGACACT	WT promoter construct (PL and PS)
mpf3	CGAAATGGCC**AG**T**TATGC**CGAAGGCAATTCCAAACAACG	Mutation for distal site
mpr4	TGCCTTCG**GCATA**A**CT**GGCCATTTCGTGGAGAAAGGAC	Mutation for distal site
mpf5	CGAATAAAAGAGG**A**C**AT**A**T**G**TGT**TACGGGACACAGTCACCCCCT	Mutation for proximal site
mpr6	CGTA**ACA**C**A**T**AT**G**T**CCTCTTTTATTCGTTCCAACAAG	Mutation for proximal site
pfA	GGGAAAGGCCATTTCGTGGAG	CHART-PCR for area A
prA	ATTGATTGGCTGCTGTGACCAT	CHART-PCR for area A
pfB	GTGCCCATCCATTTGCGAATTG	CHART-PCR for area B
prB	GGCTCGGCGCAATCTGCTGT	CHART-PCR for area B
pfC	CTCCCACGGTGAACCAACTCT	CHART-PCR for area C
prC	GCATCTGGGCTCTCAGACACT	CHART-PCR for area C
Rpf1	GGACACTGACATGGACTGAAGGAGTA	5'RACE nested
Rpf2	CGACTGGAGCACGAGGACACTGA	5'RACE
Rpr1	GGCTCCCAAGTGTAACAGAGAC	5'RACE nested
Rpr2	TGCAGGTGGGGATCAATGTGA	5'RACE

*, Mutated nucleotides are shown as bold letters.

#, Underlined are artificially introduced restriction site.

Additional file 1, Supplementary Table S3A**:** mRNA levels in Tissues.

	Liver			MG			
TFs	GS	Infected	fold induction vs GS	GS	Neighbor	Infected	fold induction vs GS
CEBP-β	49 ± 5	164 ± 41*	3.32	36 ± 6	59 ± 7	301 ± 30**	8.36
CEBP-α	832 ± 96	1509 ± 186*	1.81	85 ± 6	160 ± 10	245 ± 38**	2.88
CEBP-δ	51 ± 6	79 ± 7*	1.55	16 ± 4	43 ± 9	163 ± 26**	10.18
NFYA	409 ± 18	578 ± 64*	1.41	461 ± 65	629 ± 39	774 ± 63**	1.68
SREBP1a	881 ± 143	505 ± 39*	0.57	2538 ± 635	979 ± 122	880 ± 102*	0.35
p65	115 ± 5	215 ± 27**	1.86	117 ± 16	196 ± 11	242 ± 19**	2.07
p50	655 ± 35	939 ± 81*	1.43	1956 ± 302	2822 ± 89	5962 ± 463**	3.05

Values are mean (± S.E.M.) copy numbers relative to 75 ng of total RNA used as input for cDNA generation; MG, mammary gland; TF, transcription factor; GS or Infected, tissues gold standard or infected cows; Neighbor, sterile udder quarter neighboring an infected one.

Additional file 3, Supplementary Table S3B: mRNA levels in model cells.

	HepG2	MAC-T
TF	Mean ± SEM	Mean± SEM
SREBP1a	21 ± 9	453 ± 51
C/EBP-α	41 ± 6	173 ± 6
C/EBP-β	160 ± 10	76 ± 3
