# Supplementary material for: Stearoyl-CoA desaturase 1 expression is downregulated in liver and udder during E. coli mastitis through enhanced expression of repressive C/EBP factors and reduced expression of the inducer SREBP1A
Source: BMC Mol Biol. 2016 Jul 20;17:16. doi: 10.1186/s12867-016-0069-5 (PMC4955114; doi:10.1186/s12867-016-0069-5)
Supplement: Supplementary file 2 — 10.1186/s12867-016-0069-5 DNA sequence comparison of the SCD1 promoter from cattle and human indicating positions and motifs of relevant transcription factors and restriction sites. Figure S2. Map of the domains of wt and DN-variants of the factors C/EBPα and −β. Figure S3. Differentiation of the effect of C/EBPα and −β on the activity of the truncated short promoter. Data were achieved in identical experimental settings as shown in Fig. 5, however using the reporter gene construct PS. [file 12867_2016_69_MOESM2_ESM.pdf]

Additional file 2: Supplementary Figures

Supplementary Figure S 1: DNA sequence alignment of the SCD1 promoter from cattle and human.

```
                  WT_PL
Bovine    -1767   GGAGCAACACCTGCCCAGACTTCTCTCCCACAAACATGCTGAGGGCTTCTGAGCCGTGAT
Human               GT  GAC AC TA    T    G G     ----   CA  TA         G A   C

Bovine    -1707   GAGAC-AGCTGTCTGTATCTAAAAACATCTAGTCTCATTCAAAACTTTTGCA\······\
Human             TG  TG       CAC    G   T    C     TG     G      CA  \······\

                                                                        NFY(-)
Bovine    -1168   CTACCTACCTCACAGGGCCACA-AGTTAAGAATCAAATGAAATGATTCTTGTGGATTGGC
Human             G    C     C     T    G  ---    GC T C GA    C          A

                                                                  ScaI
Bovine    -1109   TTG-----------CAATACCTTTTGGCTACTCTTGCTGCACTTACAAGTACTTTCTCCT
Human                CAGTAATAATT        GCCA    T    AT C   A C    CC      G

                                                           NF-κB(+) C/EBP(-)
Bovine    -1060   GCTTCTGAGTGAAAGCAC--GTCTGTCCTTTCTCCACGAAATGGCCTTTCC-CAACGAAG
Human                G   G      A   AT    A  -G     CTG    --------G    A   A

                  NF-κB(+) C/EBP(-)
Bovine    -1003   GCAATTCCAAAACAACGACCCGACGTTCT----AATCTCCAAAACATCCCGC\······\
Human             ---         TT  A   T C AG   GAAG                   \······\

Bovine    -361    AGAAGCCGAG---GAGAAAGGGAGGGGAGGGGTAGTGAGGAGCTCGCGGCAGAGGGAACA
Human                   T   AAG      CA           G  C        G              A

                    SREBP(+)      NFY(+)                NF1(+)
Bovine    -304    GCAGATTGCGCCGAGCCAATGGCAACGGCAGGACGAGGTGGCACCAAATTCCCTTCGGCC
Human

                                                                  SP1(+)
                  NFY(+)                                    AP2(-)
Bovine    -244    AATGACGCGCCAGAGTCTACAGAAGCC-CATTAGCATTTCCCCAGGGGCAGGGGCAGAGG
Human                    A   G    T          T                 A           G

Bovine    -185    CAGGGGCTGCGGCGGCCAAGCCGCGGTGT--GTGT--GCAGCATCCAGTTCTTGCTTCTT
Human                A   C - T  ------T T     CG    CG         CCGG GCC   G  G

                                                           SP1(-)
Bovine    -129    CGGCCCCCAGCACGCCTCGGCGCTCTGTCTCCTCCCCTCTCCCGCCCATGC---------
Human                T G  -  GA         -               -         T A CTCCACGCG

                                                  PPRE(-)     NF1(+)
Bovine    -78     GGATCTCCCACGGTGAACCAACTCTGCGCACTTTGCCCCTTGTTGGCAACGAATAAAAGA
Human                C G   G - CC GT      CT              GC      G  G       G

                                     +1 TSP
                       C/EBP(+)
Bovine    -18     GGTCTGAGGAAATACGGGACACAGT
Human               G            C      GGT
```

The tsp, as found in this study is indicated, together with binding sites for selected transcription factors on the +/ or – strand are indicated. Nucleotides exchanged in the human sequence are given below the bovine sequence. White letters with underlay indicate terminal nucleotides of area found to be absolutely necessary for functioning of the promoter; red underlay, cattle [29]; black underlay, human [31].

Supplementary figure S 2

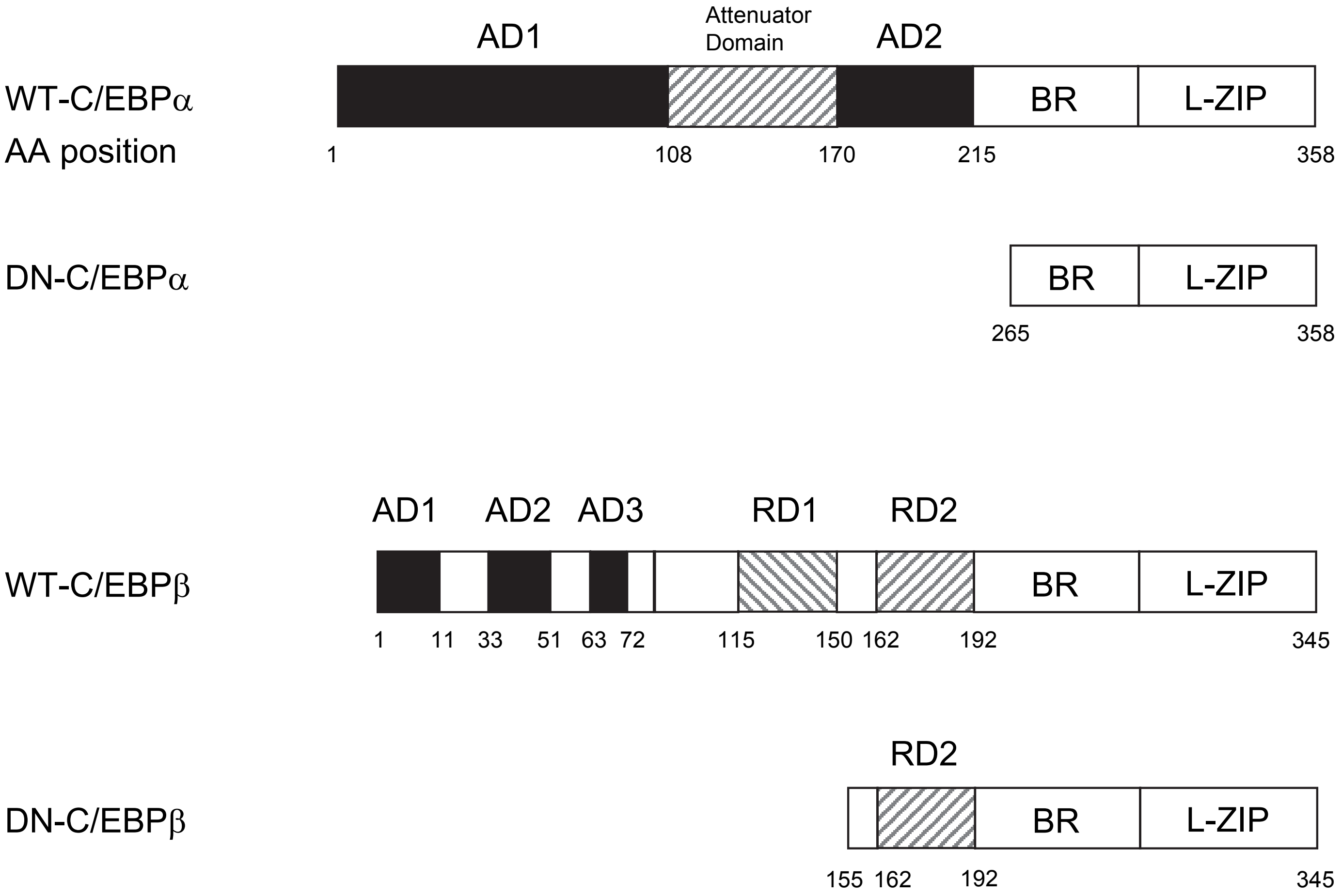

AD, RD, activator or repressive domains, respectively; BR, DNA-binding region; L-ZIP, leucin zipper for factor dimerization. Construction of the DN-factors from cattle has been described [40].
Figure has been adopted and modified from Williamson et al., 1998, Vet Immunol Immunopathol 129:36-48. AA-positions denoting the domain boundaries have been taken from the latter reference.

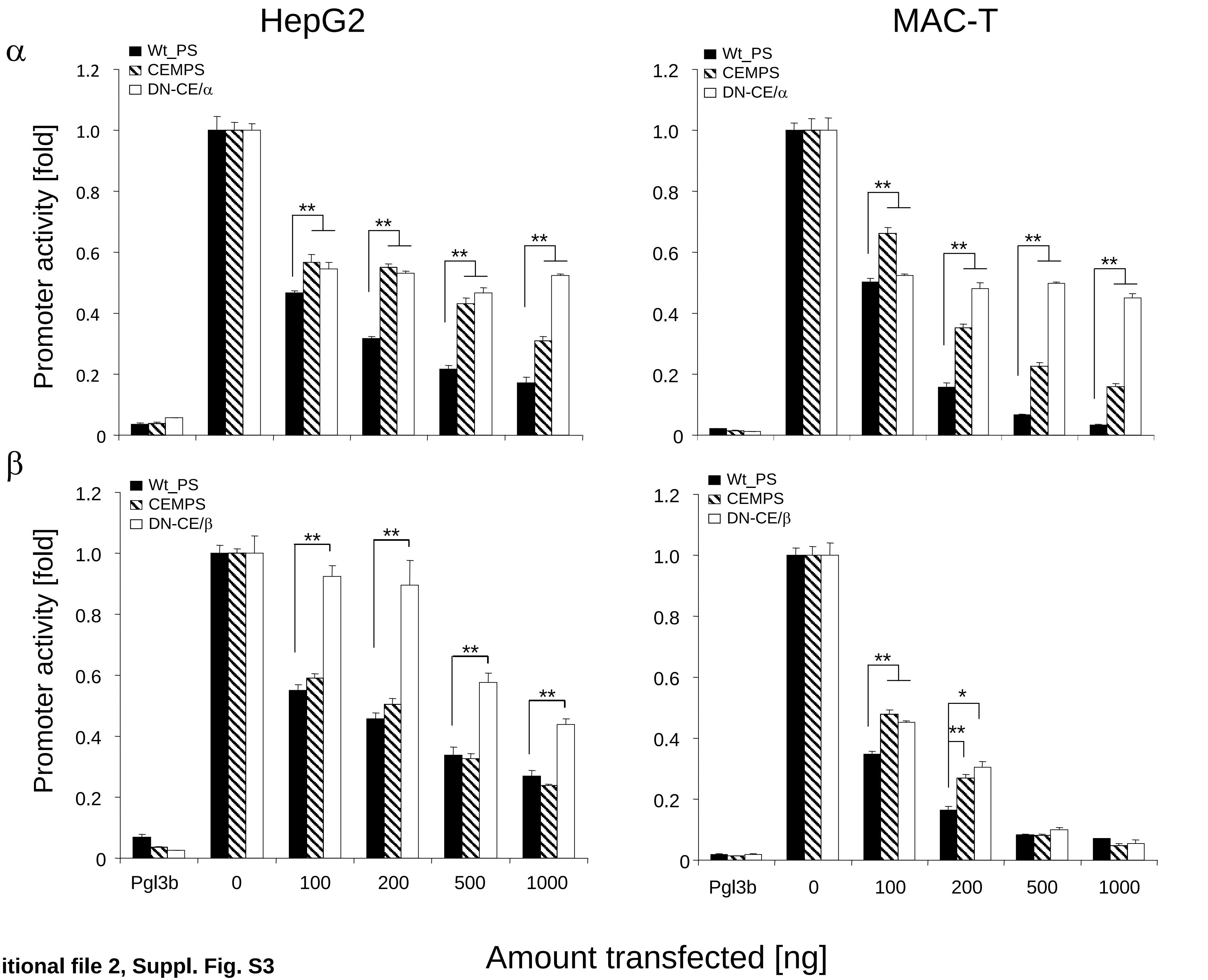

Additional file 2, Suppl. Fig. S3
